# Mechanistic Insights into the Protection Effect of Argonaute–RNA Complex on the HCV Genome

**DOI:** 10.3390/biom12111631

**Published:** 2022-11-03

**Authors:** Haiming Zhuang, Dong Ji, Jigang Fan, Mingyu Li, Ran Tao, Kui Du, Shaoyong Lu, Zongtao Chai, Xiaohua Fan

**Affiliations:** 1 Medicinal Chemistry and Bioinformatics Center, School of Medicine, Shanghai Jiao Tong University, Shanghai 200025, China; 2 Department of Anesthesiology, Changhai Hospital, Naval Medical University, Shanghai 200433, China; 3 School of Chemistry and Chemical Engineering, Shaoxing University, Shaoxing 312000, China; 4 Key Laboratory of Carcinogenesis and Cancer Invasion of Ministry of Education, Department of Liver Surgery and Transplantation, Liver Cancer Institute and Zhongshan Hospital, Fudan University, Shanghai 200032, China; 5 Department of Hepatic Surgery, Shanghai Geriatric Cancer, Shanghai 201104, China

**Keywords:** argonaute, microRNA, molecular dynamics simulations, hepatitis C virus, gene regulation

## Abstract

While host miRNA usually plays an antiviral role, the relentless tides of viral evolution have carved out a mechanism to recruit host miRNA as a viral protector. By complementing miR-122 at the 5′ end of the genome, the hepatitis C virus (HCV) gene can form a complex with Argonaute 2 (Ago2) protein to protect the 5′ end of HCV RNA from exonucleolytic attacks. Experiments showed that the disruption of the stem-loop 1(SL1) structure and the 9th nucleotide (T9) of HCV site 1 RNA could enhance the affinity of the Ago2 protein to the HCV site 1 RNA (target RNA). However, the underlying mechanism of how the conformation and dynamics of the Ago2: miRNA: target RNA complex is affected by the SL1 and T9 remains unclear. To address this, we performed large-scale molecular dynamics simulations on the AGO2-miRNA complex binding with the WT target, T9-abasic target and SL1-disruption target, respectively. The results revealed that the T9 and SL1 structures could induce the departing motion of the PAZ, PIWI and N domains, propping up the mouth of the central groove which accommodates the target RNA, causing the instability of the target RNA and disrupting the Ago2 binding. The coordinated motion among the PAZ, PIWI and N domains were also weakened by the T9 and SL1 structures. Moreover, we proposed a new model wherein the Ago2 protein could adopt a more constraint conformation with the proximity and more correlated motions of the PAZ, N and PIWI domains to protect the target RNA from dissociation. These findings reveal the mechanism of the Ago2-miRNA complex’s protective effect on the HCV genome at the atomic level, which will offer guidance for the design of drugs to confront the protection effect and engineering of Ago2 as a gene-regulation tool.

## 1. Introduction

MicroRNAs (miRNA), which are short non-coding RNAs of ~22 nucleotides, could guide Argonaute (Ago) proteins to interact with target complementary RNAs, yielding the RNA-induced silencing complex (RISC) [1,2]. The formation of the RISC can induce post-transcriptional gene silencing through translation repression or target RNA degradation. This approach plays a key role in regulation of endogenous gene expression and the defense against exogenous RNAs [3]. In targeting the exogenous RNAs from viruses, the host miRNA usually plays an antiviral role. However, the relentless tides of viral evolution have carved out mechanisms to recruit host miRNA as a viral protector [4].

Among them, Hepatitis C Virus (HCV) uniquely utilizes the liver-specific miRNA-122 for replication. MiRNA-122 could interact with the 5′ noncoding region of the HCV genome, which was the first direct viral RNA–host miRNA interaction to be discovered [5,6]. An in vitro cell assay showed that this interaction is required to maintain a high viral RNA abundance [5]. HCV infection is a leading cause of liver failure and hepatocellular carcinoma worldwide, with there being over 170 million infected individuals [7,8]. However, the current standard anti-HCV therapy with interferon-α and ribavirin maintains viral clearance in only 50% of patients and lacks specificity [8]. Thus, targeting miRNA-122 to inhibit the HCV replication could be an effective therapeutic strategy with higher specificity, and the miRNA-122-specific inhibitor Miravirsen has shown promising results against HCV infection in phase II clinical trials [8,9,10].

**Figure 1:**
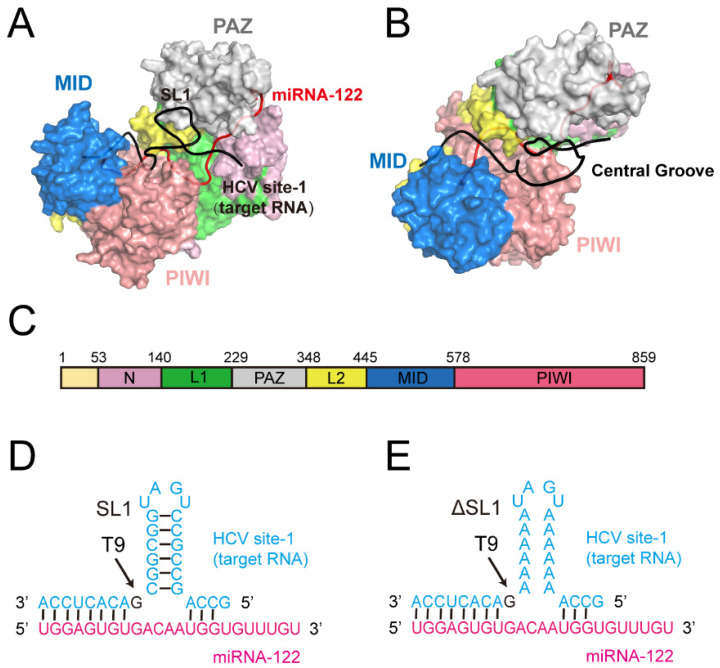
Overview of the Ago2–RNA complex. (**A**,**B**) Surface representation of the Ago2: miRNA (red): HCV site 1 RNA (black) complex. The SL1 structure and the central groove were noted. (**C**) The domain organization of the Ago2 protein. Nucleotide sequences of miR-122 (magenta) and target RNA (blue) in the WT (**D**) and the ΔSL1 (**E**) system. The T9 (G21) nucleotide (black) is indicated with arrows.

Two complementary sites (site 1 and site 2) of miRNA-122 at the very end of the HCV genome have been reported [11]. Besides the seed region and the supplementary region that is complementary to the miRNA-122, the site 1 region of the HCV genome contains a special stem-loop structure (SL1) (Figure 1). The host protein Ago2, which acts as the core of RISC, is guided by the miRNA-122 to bind with the site 1 and site 2 regions, protecting the 5′ end of HCV genome from enzyme attacks [12]. Ago2 is a bi-lobed protein with a central groove for the binding of miRNAs and target RNAs, and it is composed of four globular domains (N, PAZ, MID and PIWI) and two linker domains (L1 and L2) [13] (Figure 1). The newly reported crystal structure of the Ago2: miRNA-122: HCV site 1 (target RNA) showed that with the SL1 structure propping up the central cleft between the PAZ domain and the PIWI domain, the Ago2 protein adopted an extended conformation [14]. The crystal structure also showed that the 9th nucleotide of the target RNA (T9, guanine) is flipped out and bound in a hydrophobic pocket of Ago2, which was observed for the first time. For the special structure of the SL1 and T9, experiments have shown that the disruption of the SL1 by complete nucleotide mutation to adenine or the depletion of the basic group of the T9 could increase the Ago2 affinity with a lower Ago2-off rate [14], indicating that the SL1 and T9 are detrimental to Ago2 binding. This seems to be contradictory to the fact that the SL1 and T9 are highly conserved in the HCV genome.

Although research has shown that this might be related to the unique mechanism of the exonuclease involved in the target RNA degradation [14], the underlying mechanism of how the conformation of Ago2: miRNA: HCV site 1 is affected by the SL1 and T9 remains unclear. Furthermore, although there are many studies on the assembly of the Ago2: miRNA: target RNA complex, the structural basis of how Ago2-miRNA interacting with the target is still poorly understood [15,16,17]. Thus, with the special structure of the SL1 and T9, the Ago2: miRNA: HCV site 1 complex is a good entry point to study the inner interaction of the Ago–RNA complexes. Additionally, although the crystal structure of the Ago2: miRNA: HCV site 1 complex has been solved, it is challenging to provide enough information for the conformational dynamics of the ternary complex with only one averaged snapshot. A molecular dynamics (MD) simulation, which explores the protein and nucleic acid conformation at the dynamic and atomic levels [18,19,20,21,22], is helpful to investigate the protein binding pocket [23,24] and protein-DNA/RNA recognition [25]. In recent works, we have used the MD simulation to illuminate the structural mechanism of the protein–protein or protein-nucleic acid interactions [26,27,28,29,30]. Previous studies on the Ago2 complexes utilizing the MD simulation have contributed to the understanding of the mechanism of the Ago2–RNA interaction [31,32,33].

Here, we performed an extensive large-scale MD simulation to investigate how the T9 and SL1 structures of the HCV site 1 RNA affect the target RNA binding affinities. The results showed that the T9 and SL1 structures could induce the departing and less coordinated motion of the PAZ, PIWI and N domains, propping up the mouth of the central groove, which in turn causes the target RNA to be liable to dissociate. Moreover, combining with previous studies, we proposed a new model that the Ago2 protein could adopt a more constraint conformation with the proximity to and the more correlated motion of the PAZ, N and PIWI domains to protect the target RNA from dissociation. This study aims to shed light on the mechanistic understanding of the role of the T9 and SL1 structures, inspiring further research on developing anti-HCV strategies and engineering Ago proteins as gene regulation tools.

## 2. Materials and Methods

### 2.1. Structure Preparation

Three systems were performed with the same Ago2: miRNA-122 complex and different target RNAs, including the wild-type target RNA (denoted as “WT”), the T9 base pair-depleted target RNA (denoted as “T9-abasic”) and the SL1-disrupted target RNA (denoted as “ΔSL1”). The SL1 disruption was achieved by the mutation of all of the paired nucleotides in the stem-loop to adenine. The RNA sequences in the three systems are shown in Figure 1D,E. The crystal structure of the Ago2: miRNA-122: HCV site 1 was selected as the initial structure (PDB ID: 7KI3) [14]. The missing nucleotides were added based on the structure of the Ago2–RNA complex with the PDB ID 4Z4D [34], and the missing residues of the Ago2 protein was added using the MODELLER program. Ten thousand steps of minimization were then performed using Discovery Studio 2019 by the steepest descent algorithm to optimize the complex structure. The minimized structure was selected as the WT system. For the T9-abasic system, the base of T9 was depleted using Discovery Studio 2019, and the non-standard force field of the abasic site was built utilizing the Antechamber program of Amber and the Generalized Amber Force Field. The mutations of the nucleotides in the ΔSL1 system were performed using Discovery Studio 2019.

### 2.2. MD Simulation

The systems were prepared with the LEaP program using ff14SB and “Rochester” torsions force field to describe the RNA–protein complexes [35,36,37,38]. The ternary complex was solvated into a truncated octahedron transferable intermolecular potential three-point (TIP3P) water box [39], and Na+ and Cl- were added to simulate a normal saline environment. Two rounds of minimization were performed. The first round contained 5000 steps maximum minimization cycles with the complex being fixed, and the second round contained 10,000 steps maximum minimization cycles with no constraints. Subsequently, all of the systems were heated from 0 to 300 K within 300 ps, which was followed by 700 ps equilibration running in a canonical ensemble (NVT). After all of the preparations were conducted, 5 replicas of independent 1 μs simulations were performed under random velocities under isothermal isobaric (NPT) conditions. For the long-range electrostatic interactions, the particle mesh Ewald method [40] was used. Covalent bonds involving hydrogens were constrained using the SHAKE method [41].

### 2.3. Principal Component Analysis (PCA)

The principal component analysis (PCA), which is widely used in describing the kinetic processed that occur during simulations, can transform a series of potentially coordinated observations into orthogonal vectors. Among the vectors, the first two principal component (PC1 and PC2) provide the dominant motions during the simulation [42]. In this analysis, the PCs were generated based on the coordinate covariance matrix of the Cα atoms in the Ago protein every 10 frames, and the collected frames were all projected onto the first and second PCs.

### 2.4. Generalized Correlation Analysis

Generalized correlation (*GC_ij_*) analysis was applied to calculate the correlated motion. Compared to the traditional Pearson correlation analysis, the *GC_ij_* analysis is independent of the mutual orientations of the atomic fluctuations and can distinguish between the linear and nonlinear contributions [43]. Mutual Information (*MI*) was introduced to reflect how much information of one atom’s position was provided by another:

(1) $$MI\left[x_{i},x_{j}\right]=\int^{\;}\int^{\;}p\left(x_{i},x_{j}\right)\ln\frac{p\left(x_{i},x_{j}\right)}{p\left(x_{i}\right)p\left(x_{j}\right)}dx_{i}dx_{j}$$

The right side of the equation can be related to the more widely known measure of entropy which calculated by:

(2) $$\;H\left[x\right]=\int^{\;}p\left(x\right)\ln p\left(x\right)dx$$

To calculate based on the correlation between the pairs of atoms:

(3) $$MI\left[x_{i},x_{j}\right]=H\left[x_{i}\right]+H\left[x_{j}\right]-H\left[x_{i},x_{j}\right]$$

*MI*[*x_i_*,*x_j_*] was further related to a more intuitive Pearson-like correlation coefficient *GC_ij_*, which can be calculated by:

(4) $$GC_{ij}={\left\{1-e^{-\frac{2MI\left[x_{i},x_{j}\right]}{d}}\right\}}^{-\frac{1}{2}}$$

where *d* represents the dimensionality of *x_i_* and *x_j_*, which is 3 in this study. The *GC_ij_* calculation was applied using g_correlation tool in Gromacs 3.3 [44], with the coordinates of Cα in each residue. The inter-domain correlation was calculated by the accumulation of the *GC_ij_* value between each residue in the respective domains. The accumulated value was normalized based on the maximum and minimum values in the counterpart in all of the three systems.

### 2.5. Dynamic Network Analysis

A network analysis was performed to reflect the motion connection using VMD [45]. In our analysis, the Cα atoms in the Ago2 protein, the N1 atoms in the uracil and cytosine, the N9 atoms in the adenine and guanine and the P atom in the abasic site were chosen as the nodes to represent their corresponding residues. The edges were drawn between the nodes whose distances were within a cutoff of 4.5 Å for at least 75% of the simulation time. The edge was calculated by:

(5) $$d_{i,j}=-\log\left(\left|C_{i,j}\right|\right)$$

where *i* and *j* represent the two nodes. Additionally, community, which means a group of residues whose connections are stronger was calculated using the Girvan–Newman algorithm [46] through the gcommunities program of VMD.

### 2.6. Markov State Model (MSM)

The Markov state model (MSM) is a mathematical framework that is used to describe the dynamics of time-series data [47,48,49,50]. In our analysis, the MSM was used to differentiate between the conformational states of the energy basin map and to help to extract representative structures from each state. All of the MSM calculations were performed using the PyEMMA software 2.5.7. The MSM transition matrix was first calculated based on the probability of the transition between the different states.

An implied timescale (ITS) test was performed to check the Markovian property and select the proper lag time (*τ*). The ITS as a function of *τ* can be calculated by:

(6) $$t_{i}=-\frac{\tau }{\ln\left|\lambda_{i}\left(\tau \right)\right|}$$

where *λ_i_* means the eigenvalue which was obtained from the MSM transition matrix of the *i*th process. The implied timescale plot can be drawn in which each curve represents the average transition time of one process. When the curve became approximately constant, the corresponding lag time was chosen for the following analysis and the system had a Markovian property. In this study, the lag time was set as 1 ns.

Every frame of each system was projected on the free energy plot according to the characteristic vectors. Then, the K-means algorithm was applied to cluster the two-dimensional conformations into 300 microstates in each system. Based on the 300 microstates, a PCCA+ algorithm was performed to divide them into different clusters. With the divided clusters, the Chapman–Kolmogorov test was applied to further testify the property of the MSM. The structure with the smallest root-mean-square deviation (RMSD) with the frames in the same cluster was chosen as the representative structure.

## 3. Results

### 3.1. The Disruption of T9 and SL1 Affects Conformational Dynamics of Ago2–RNA Complex

A total of three systems with different target RNAs were performed, one with the wild-type HCV site 1 RNA (WT), one with the T9 base pair depletion target RNA (T9-abasic), and the other with the SL1-disruption target RNA (ΔSL1). The RNA sequences of the three systems are shown in Figure 1D,E. To investigate the overall conformational dynamics in the three systems, we calculated the Cα atoms root-mean-square deviation (RMSD) of the Ago2 protein relative to the initial structure. The RMSD reflects the amplitude, but not the direction of the variation of the simulated structures relative to the initial structure. The results indicated that the three systems reached equilibrium after 200 ns of the simulation (Supplementary Figure S1), and the following analyses were all based on the frames in equilibrium. The RMSD values were 2.06 ± 0.30 Å for the WT system, 2.29 ± 0.42 Å for the T9-abasic system and 2.00 ± 0.21 Å for the ΔSL1 system.

**Figure 2:**
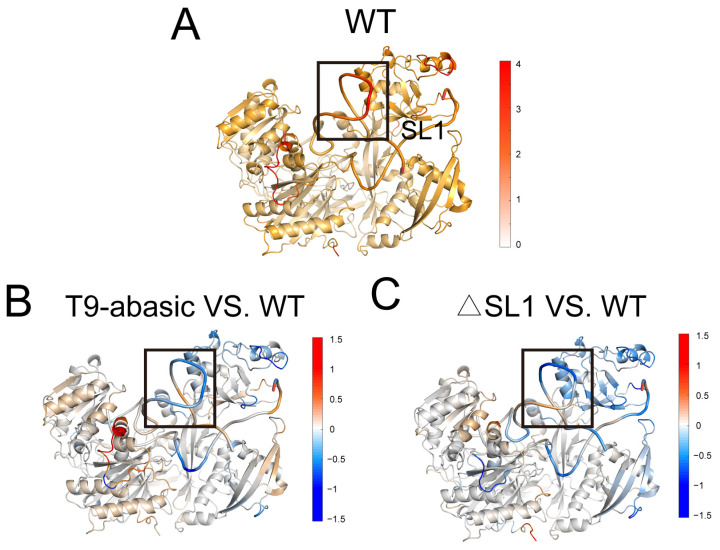
Conformational dynamics of the Ago2–RNA complex. (**A**) The averaged RMSF values of each residue are plotted on the 3D structures of the Ago2 protein in the WT system. The substations of RMSF values of the T9-abasic and ΔSL1 systems from the counterpart of the WT systems are plotted on the 3D structures (**B**,**C**). Positive regions (red) stand for higher RMSF values, whereas negative regions (blue) represent lower RMSF values. The significant fluctuation differences are highlighted with a dashed rectangle in each panel.

To reflect the local dynamics in the Ago2–RNA complex, the root-mean-square fluctuation (RMSF) of each residue (represented by the Cα atoms for Ago2 and P atoms for RNA) was calculated (Supplementary Figure S2). To better show the differences, we subtracted the RMSF value of the T9-abasic and the ΔSL1 system from its counterpart in the WT system and projected the absolute value of the WT system and the difference values of the T9-abasic and the ΔSL1 system on the protein structure (Figure 2). The inspection of the RMSF plot showed that the SL1 region displayed the highest fluctuation in the WT system, and its RMSF value significantly dropped in the T9-abasic and the ΔSL1 system, indicating that the disruption of T9 and SL1 could stabilize the stem-loop region of the target RNA. In addition, the global fluctuation of the PAZ and PIWI domains generally decreased with the disruption of T9 and SL1, which may be correlated with the tension release of the central groove of Ago2. All of these findings were in line with the fact that compared to the WT system, the disruption of T9 or SL1 could increase the Ago2 affinity to the target.

### 3.2. The Disruption of T9 and SL1 Induces the Conformational Transition of Ago2

**Figure 3:**
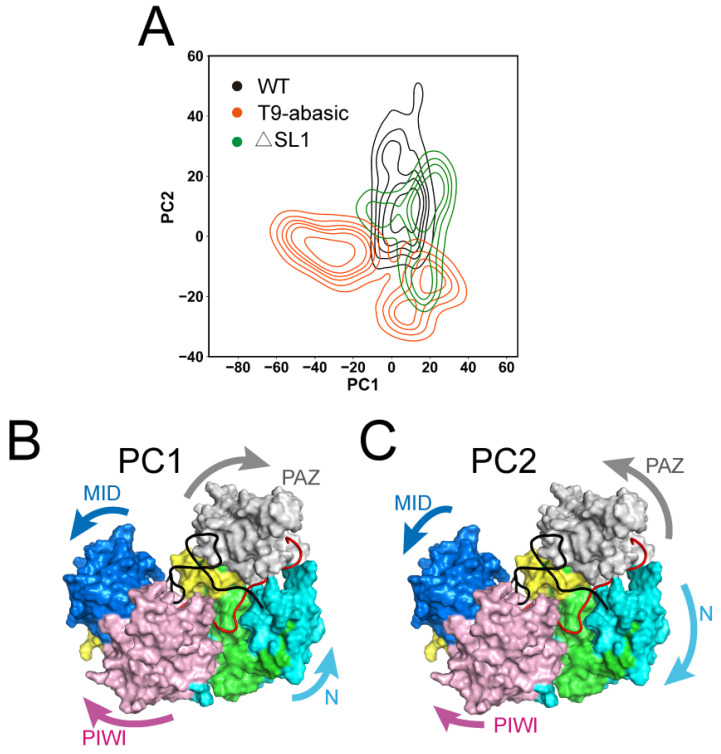
Global conformational transitions of the Ago2–RNA complex. (**A**) Projections of the first and second principal components (PC1 and PC2) from MD simulations of the Ago2 protein in three systems. The domain motion along PC1 (**B**) and PC2 (**C**) was shown.

To characterize the global conformational transition in the three systems, a principal component analysis (PCA) was performed (see in Materials and Methods). We projected the snapshots every 10 frames throughout the simulation of each system on the two-dimensional plot according to the first two principal components (PC1 and PC2) (Figure 3A), and the domain motion along the PC1 and PC2 was shown on the Ago2 protein (Figure 3B,C).

The T9-abasic system contains one cluster of conformations with a PC1 value that is less than −10, which was not observed in the WT and ΔSL1 system, indicating a wider conformational space that was induced by the T9 base pair depletion. Along the PC1, the PAZ and N domains underwent a departing motion away from the MID and PIWI domains, widening the central groove transversely. The motion suggested that the Ago2 protein adopted a more constrained conformation in the T9-abasic system. Compared with the WT system, the PC2 value decreased in the T9-abasic system and the ΔSL1 system. Along the PC2, the PAZ and PIWI domains moved away from each other and drove the MID and PIWI domains closer together, yielding the longitudinal opening of the central groove which accommodates the target RNA. This indicates that the disruption of the T9 and SL1 structure could fasten the mouth of the central groove, thus, protecting the target RNA from dissociation. Taking the information provided by PC1 and PC2, we can conclude that the WT T9 and SL1 structures could induce the Ago2 protein in a more extended conformation, leading to a more opening mouth of the central groove which accommodates the target RNA. The opening of the mouth of the central groove could make the target RNA liable to dissociate, which is in line with the experiment results that the T9 or SL1 could decrease the Ago2 affinity to the target RNA.

**Figure 4:**
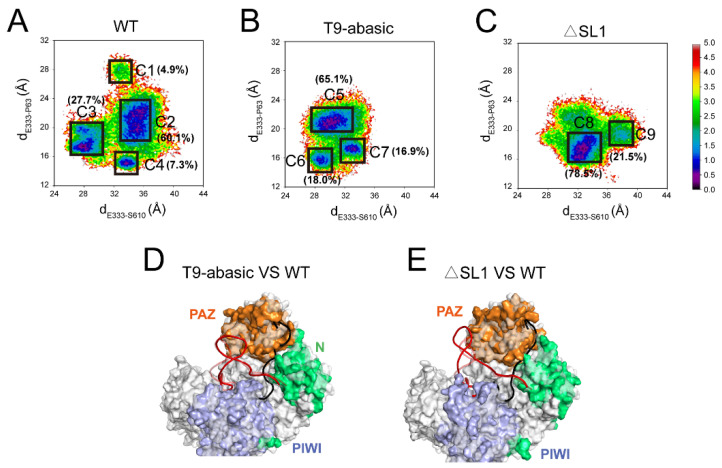
The Ago2 protein adopted a more constrained conformation in the T9-abasic and ΔSL1 systems. (**A**–**C**) Conformational landscapes which were generated using the dE333–S610 (distance from the E333 Cα atom to the P610 Cα atom) and dE333–P63 (distance from the E333 Cα atom to the P63 Cα atom) parameters in the WT system (**A**), the T9-abasic system (**B**) and the ΔSL1 system (**C**). Superposition of the dominant conformations from the T9-abasic system (**D**) and the ΔSL1 system (**E**) to the WT system (transparency), respectively.

To further prove the conformational transition that was induced by the T9 and SL1 structure, we projected all of the MD trajectories onto the two-dimensional landscape according to the distances from the E333 Cα atom to the S610 Cα atom and from the E333 Cα atom to the P63 Cα atom (Figure 4A–C). D_E333–S610_ represents the distance between the PAZ and PIWI domains, while d_E333–P63_ represents the distance between the PAZ and N domains (Supplementary Figure S3). Thus, the d_E333–S610_ and d_E333–P63_ can reflect the opening of the central groove. The Markov state model (MSM) was used to differentiate the characterized clusters in the free-energy landscape (see in Material and Methods). To testify the Markovian property, the implied timescale test and the Chapman Kolmogorov test were applied (Supplementary Figure S4). The proportion and the representative structures of each state were calculated based on the MSM.

Four states were observed in the WT system with the decrease in the d_E333-P63_ parameter: C1, C2, C3 and C4, and the C2 state was the dominant conformation with it having a proportion of 60.1%. In the T9-abasic system, three clusters (C5, C6 and C7) were observed with the dominant conformation of C5. In the ΔSL1 system, with the increase in the d_E333–S610_ parameter, the dominant C8 state and the minor C9 state were observed. Compared with the WT systems, the states in the T9-abasic system had relatively lower d_E333–S610_ and d_E333–P63_ parameters, indicating that there was a shorter distance between the PAZ, N and PIWI domains. The d_E333–P63_ parameter was reduced in the ΔSL1 system when it was compared with the WT system, reflecting a closer relationship between the PAZ and N domains, which was in accordance with the motion along PC2. In general, the landscape proved the conformational change that was revealed in the PCA analysis and suggested a more proximal relationship between the PAZ, N and PIWI domains with the disruption of the T9 and SL1 structure.

To further illustrate the conformational transition, we superimposed the representative structures of the dominant state in the T9-abasic system (C5) and the dominant state in the ΔSL1 system (C8) with the dominant state in the WT system (C2), respectively (Figure 4D,E). Though with the mutation to disrupt the SL1 structure in the ΔSL1 system, a stem-loop like structure was preserved during the simulation. This was ascribed to the limited simulation time. To better reflect the conformational changes, the structure of the WT was set to it being transparent. An inspection of the superimposition showed that the proximal of the PAZ, N and PIWI domains that were induced by the disruption of the T9 and SL1 structure caused the shrinkage of the mouth of the central groove, which fastened the target RNA-binding pocket. Taking the data together, the analysis suggested that the WT T9 and SL1 structures could induce a distant relationship between the PAZ, N and PIWI domains, propping up the mouth of the central groove which acts as the target RNA-binding pocket, which in turn makes the target RNA liable to dissociate.

### 3.3. The Disruption of T9 and SL1 Disturbs the Correlated Motions and Community Networks of the Ago2–RNA Complexes

**Figure 5:**
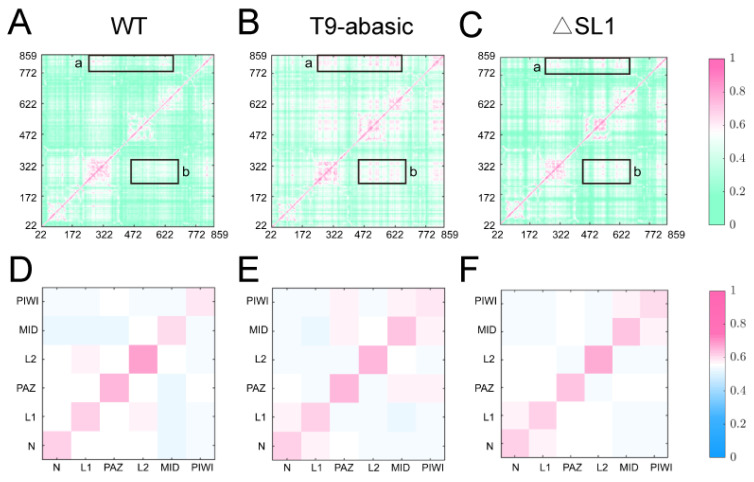
Inter-residue and inter-domain correlation of the Ago2 protein. Generalized residue correlation of the WT system (**A**), the T9-abasic system (**B**) and the ΔSL1 system (**C**). The significant correlation differences are highlighted with a rectangle in each panel. Generalized domain correlation of the WT system (**D**), the T9-abasic system (**E**) and the ΔSL1 system (**F**).

Using a generalized correlation analysis, an overview of the inter-residue and inter-domain-correlated motions was provided (see in Material and Methods). The results showed that the inter-residue correlation of regions A and B were significantly strengthened with the disruption of the T9 and SL1 (Figure 5A–C). The region a represents the interaction between the residues which are from the PIWI domain and close to the central groove and the residues from the PAZ and MID domains, and thus, its reinforcement suggested that the PIWI domain having a more correlated motion with the PAZ and MID domains is induced by the disruption of the T9 and SL1 structure. Similarly, the enhancement in the region b reflected a more coordinated motion between the residues close to the central groove from the PAZ, PIWI and MID domains. In accordance with the inter-residue correlation, the investigations on the interdependent motions between the different domains also proved that the correlated motion between the PAZ, PIWI and MID domains was strengthened in the T9-abasic system when it was compared with the WT system (Figure 5D,E). However, the variation between the ΔSL1 system and the WT system was not obvious (Figure 5F), which may be related to the confounding influence introduced by the residues that were far from the central groove in each domain.

**Figure 6:**
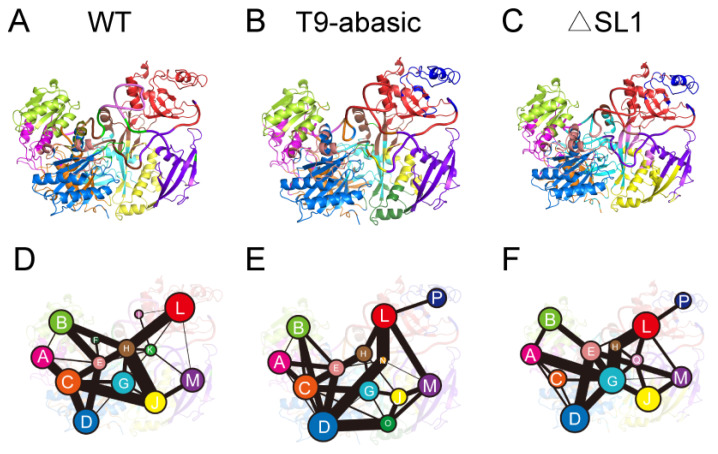
Community network of the Ago–RNA complex. Community composition in the WT system (**A**), the T9-abasic system (**B**) and the ΔSL1 system (**C**). The community network in the WT system (**D**), the T9-abasic system (**E**), and the ΔSL1 system (**F**). Areas of the circles represent the numbers of residues in corresponding communities, and the widths of sticks connecting communities represent the intercommunity connections, respectively.

A community network analysis was performed to further uncover the network organization of the Ago2–RNA complex. Each community is shown as circles whose area is proportional to the number of residues that it contains. The strength of the inter-community correlation is represented by the width of the sticks connecting the communities. The community composition and connections are shown in Figure 6. For the constitution of the communities, the SL1 structure exists as a separate community in the WT system, while in the T9-abasic and ΔSL1 system, it belongs to the same community with the PAZ domain, indicating a more correlated relationship between the SL1 and PAZ domain in the T9-abasic and ΔSL1 system, which may strengthen the interaction between the target RNA and Ago2 protein. For the correlation between the communities, the connection between Community L (mainly composed of the PAZ domain) and Community M (mainly composed of the N domain) was intensified in the T9-abasic and the ΔSL1 systems when it was compared with that of the WT system, suggesting a more coordinated motion between the PAZ and N domains which was induced by the disruption of T9 and SL1.

In conclusion, the disruption of the T9 and SL1 structure induced a more correlated motion between the residues, nucleotides and domains (PAZ, PIWI and N) around the mouth of the central groove, which may be the cause of the shrinkage of the target RNA-binding pocket which was induced by the disruption of the T9 and SL1 structure.

## 4. Discussion

The Ago2 protein, as the core of RNA-induced silencing complex, plays a critical role in regulating gene expression and defending against exogenous pathogens. However, HCV has evolved a special mechanism which utilizes the complementary sites at the 5′ end of the genome to recruit the Ago2–miRNA complex to defend against the enzyme attacks. Here, using large scale MD simulations, we investigated how the special structures, T9 and SL1, in the site 1 HCV RNA affect the target RNA recognition at the atomic level, thereby providing guidance for the development of anti-HCV strategies and the engineering of Ago2 as a gene regulation tool.

The T9 and SL1 could promote the Ago2 protein to adopt a more extended conformation. The PCA analysis and MSM analysis showed that the T9 and SL1 induce the departing motion of the PAZ, PIWI and N domains, propping up the mouth of the central groove which accommodates the target RNA, which may in turn make the target RNA liable to dissociate. Interestingly, we previously found a similar conformational change in another Ago2–RNA complex. To explore the underlying mechanism of how the miRNA extensions increased the target RNA affinity [51], we performed a series of MD simulations on the Ago2: miRNA-122: target RNA complex [52]. The simulation showed that the extension of the miRNA lengths could promote the proximity of the PAZ, PIWI and N domains to tighten the mouth of central groove, which in turn protected the target RNA from water solvent attacking and hindered the target RNAs from escaping from the Ago2–RNA complexes. Moreover, the sum of hydrogen bonds formed between target RNA and the Ago2: miRNA-122 complex was similar with different miRNA lengths. Therefore, we proved that the conformational change, rather than the enhanced interaction is the key factor for the increased the target affinity with the miRNA extensions. In this study, we also calculated the number of hydrogen bonds of the target RNA to Ago2 and miRNA in the three systems (Supplementary Figure S5), respectively. In agreement with the results in the previous study, no significant difference was observed between the three systems in addition to the slightly increased hydrogen bonds between the target RNA and the protein in the ΔSL1 system which might be ascribed to the disruption of the inner hydrogen bonds. All of these results indicate that the conformation Ago2 which was adopted played a significant role in catching the target RNA. A more constricted Ago2 conformation which means the shrinkage of the mouth of the central groove would benefit the target RNA binding.

The T9 and SL1 could affect the dynamics and correlated motion of the Ago2–RNA complexes. The RMSF analysis revealed that the SL1 region, PAZ and PIWI domains displayed a higher fluctuation in the WT system when they were compared with those of the T9-abasic and ΔSL1 systems, implying that the instability was induced by the T9 and SL1 structures. According to the GCCM and network analysis, the T9 and SL1 structures also weakened the correlated motion between the PAZ, PIWI and N domains which encompassed the mouth of the central groove. This may be related to the extended conformation which was induced by the SL1 and T9-abasic system.

The modification of the Ago proteins into gene regulation and even editing tools has been a research hotspot [53,54,55]. However, the structural basis for the recognition and assembly of the Ago–nucleic acid complexes needs to be further investigated for the development of new engineering strategies [56]. Based on the previous and current studies, we proposed a new model that the Ago2 protein could adopt a more constraint conformation with the increased proximity to and more correlated motion of the PAZ, N and PIWI domains to protect the target RNA from dissociation. Thus, the modification of the residues to intensify the interaction between the PAZ, PIWI and N domains may be a feasible strategy to increase Ago’s binding affinity.

HCV infection is liable to become chronic due to it having multiple mechanisms of resistance to clearance [57], one of which is the recruitment of Ago–miRNA complexes through the site 1 sequence to protect the terminal of its genome. Our study elucidates the role of the site 1 key structures T9 and SL1 in the conformational dynamics and assembly of the Ago2: miRNA-122: HCV site 1 complex, paving the way for the development of new anti-HCV therapies through the miR-122 pathway.

In summary, the collective sampling of the 15 μs MD simulations showed that the T9 and SL1 structure of the HCV site 1 RNA could induce the departing motion and the weakened correlation between the PAZ, PIWI and N domains, propping up the mouth of the central groove, which in turn causes the instability of the target RNA. This was in line with the experimental results that the T9 and SL1 structures were detrimental to Ago2 binding.

Moreover, we proposed a new model that the Ago2 protein could adopt a more constrained conformation with the increased proximity to and more correlated motion of the PAZ, N and PIWI domains to protect the target RNA from dissociation. Thus, the modification of the residues to intensify the interaction between the PAZ, PIWI and N domains may be a feasible strategy to increase Ago’s binding affinity. These results shed light on the structural details of the assembly of the Ago–RNA complexes, which will help to offer guidance for the development of anti-HCV strategies and the engineering of Ago2 as a gene regulation tool.

## Supplementary Materials

The following supporting information can be downloaded at: https://www.mdpi.com/article/10.3390/biom12111631/s1, Figure S1: Time evolution of the root-mean-square deviation (RMSD) of Ago2; Figure S2: Root-mean-square fluctuations (RMSF) of Ago2-RNA complex; Figure S3: The representation of dE333–V610 and dE333–P63 in the energy landscape; Figure S4: The MSM timescale and Chapman Kolmogorov tests; Figure S5: The number of hydrogen bonds of the target RNA with the Ago2 protein and miRNA.
