# Supplementary material for: Mechanistic Insights into the Protection Effect of Argonaute–RNA Complex on the HCV Genome
Source: Biomolecules. 2022 Nov 3;12(11):1631. doi: 10.3390/biom12111631 (PMC9687641; doi:10.3390/biom12111631)
Supplement: Supplementary file 1 [file biomolecules-12-01631-s001.zip › biomolecules-1969889-supplementary.pdf]

Supporting Information

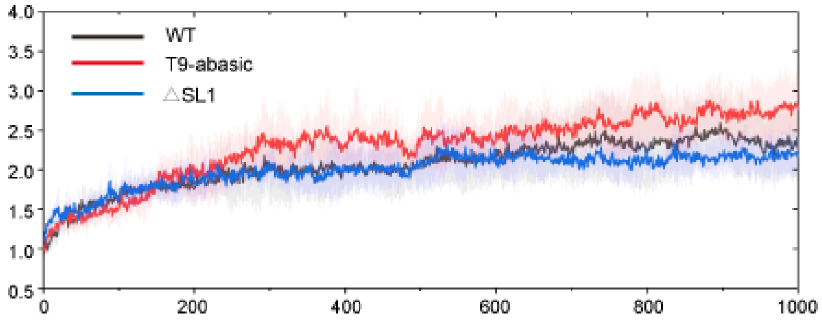

Figure S1. Time evolution of the root-mean-square deviation (RMSD) of Ago2 backbone atoms with respect to the initial structure

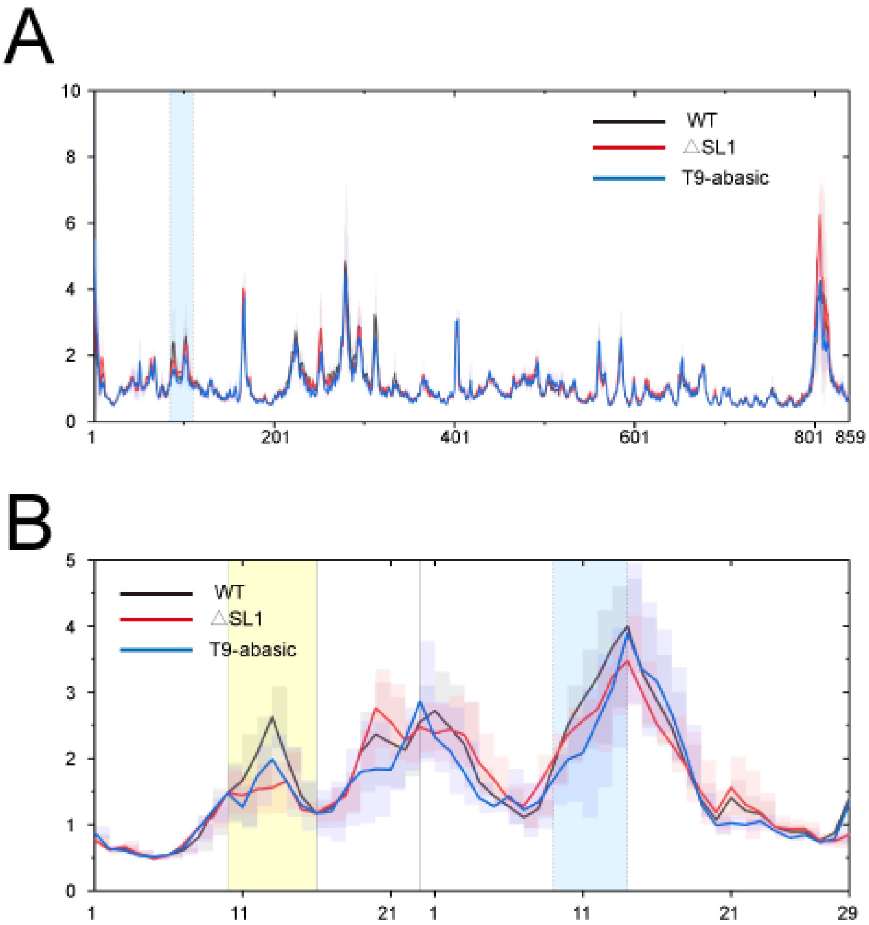

Figure S2. (A) Root-mean-square fluctuations (RMSF) of Ago2 Cα atoms averaged over 5 independent runs in the three systems. The significant fluctuation differences are marked with blue backgrounds. (B) RMSF of RNA P atoms averaged over 5 independent runs in the three systems. The significant fluctuation differences are marked with yellow and blue (represents the SL1 region) backgrounds.

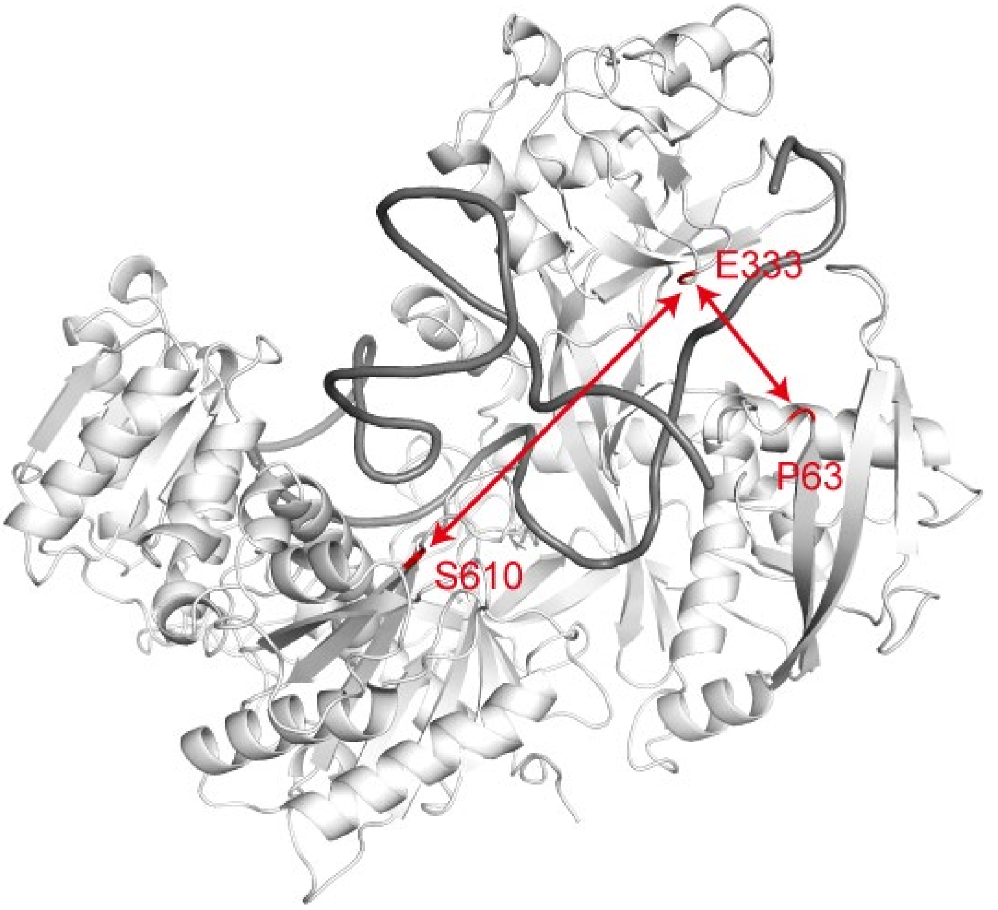

Figure S3. $D_{E333\text{-}V610}$ represents the distance between the PAZ and PIWI domains while $d_{E333\text{-}P63}$ represents the distance between PAZ and N domains.

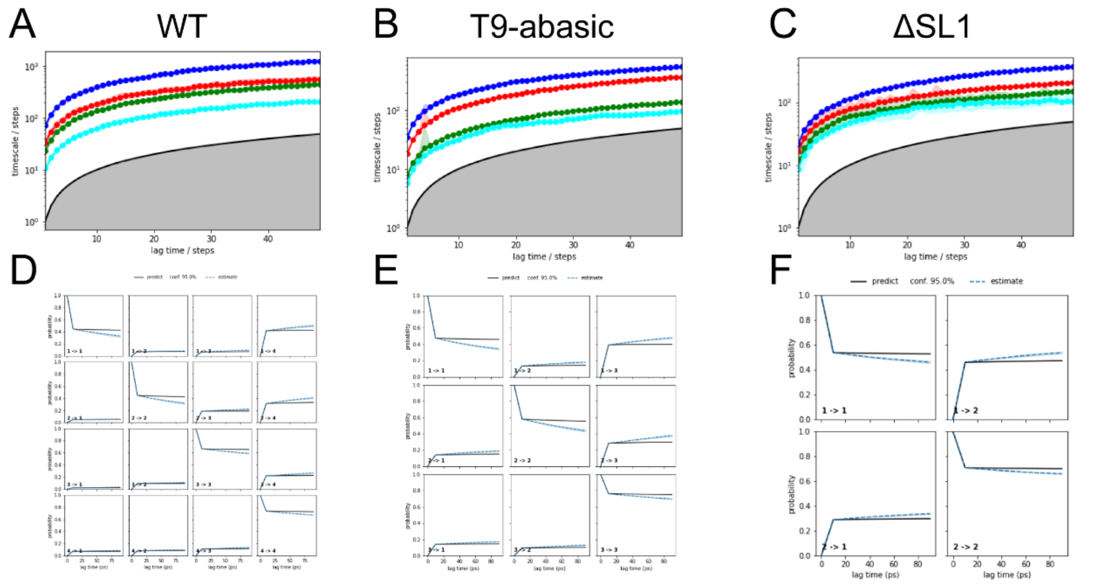

Figure S4. The MSM timescale test in the WT system (A), the T9-abasic system (B) and the ΔSL1 system (C). The MSM Chapman–Kolmogorov test in the WT system (D), the T9-abasic system (E) and the ΔSL1 system (F).

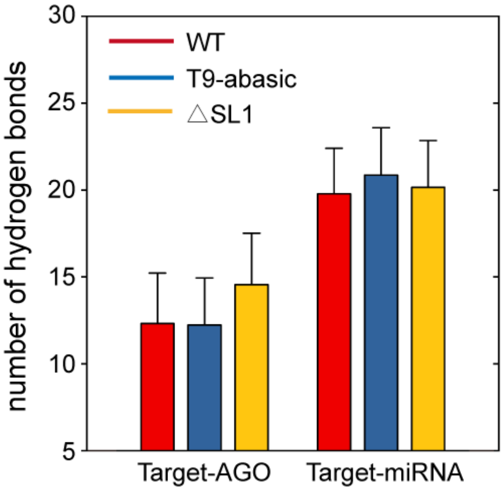

Figure S5. The number of hydrogen bonds of the target RNA with the Ago2 protein and miRNA.
